# Cervical Lymphadenopathies as Unusual Presentations of Erdheim–Chester Disease: The Need for Knowledge for Diagnosis and Treatment

**DOI:** 10.3390/life11111116

**Published:** 2021-10-20

**Authors:** Raúl Antúnez-Conde, Carlos Navarro Cuéllar, Santiago Ochandiano, Alberto Díez-Montiel, Pablo Montes, Eduardo Monteserín, Marc Agea, Dafne Gascón, Ignacio Navarro, Gema Arenas, Manuel Tousidonis, José Ignacio Salmerón

**Affiliations:** Oral and Maxillofacial Surgery Department, Hospital General Universitario Gregorio Marañón, C/Dr. Esquerdo, 46, 28007 Madrid, Spain; sochandiano@hotmail.com (S.O.); diezmontiel@gmail.com (A.D.-M.); pmontesfm@gmail.com (P.M.); eduardomonteserin@gmail.com (E.M.); ageamarc@gmail.com (M.A.); dgasconalonso@gmail.com (D.G.); nnavcu@hotmail.com (I.N.); gema.arenas@gmail.com (G.A.); manuel@tousidonisrial.com (M.T.); jisalmeron@telefonica.net (J.I.S.)

**Keywords:** immunohistochemistry, lymph nodes, head and neck, image-guided biopsy

## Abstract

The appearance of cervical adenopathies can occur in many pathologies in a non-specific manner; Erdheim–Chester disease (ECD) is characterized by xanthogranulomatous and xanthomatous infiltration of different tissues with numerous foamy histiocytes. Bone lesions are frequent and radiological features are pathognomonic for diagnosis, but lymph node involvement is exceptional and is not a form of presentation reported in the literature. Recurrent BRAFV600E mutation and others have been discovered in recent years. Since then, several treatments targeting the BRAF and MEK pathways have been developed with high success rates; even so, interferon-α continues to be one of the most widely used treatments. The best imaging test for the study and monitoring of the disease is PET-CT. The prognosis of ECD is relatively poor, with a survival of 43% of patients after 32 months follow-up. Higher survival rates have been reported in patients treated with interferon. The authors present an exceptional case of ECD with cervical adenopathies as a debut, highlighting the need for the knowledge of the disease for differential diagnosis, early treatment, and the importance of communication between the clinician and the pathologist. The main features of the disease and a brief discussion of current diagnosis and treatment are reviewed.

## 1. Introduction

Erdheim–Chester disease (ECD) was described for the first time in 1930 as a “lipoid granulomatosis” [1]. This disease is characterized by xanthogranulomatous and xanthomatous infiltration of different tissues with numerous spumous histiocytes, surrounded by fibrosis in most cases [2]. To date, approximately 1000 cases have been described in the scientific literature, demonstrating the disease oddity. It is listed as a rare disease by the European Rare Disease Organization and the National Organization for Rare Disorders [3].

The radiological characteristics are pathognomonic for diagnostics. In fact, the diagnosis of ECD depends on the combination of image features confirmed with histopathologic findings [4]. Lymph node involvement is exceptional and is not a form of presentation reported in the literature. Recurrent BRAFV600E mutation and others have been discovered in recent years. Since then, several treatments targeting the BRAF and MEK pathways have been developed with high success rates [1,5].

## 2. Case Presentation

A 36-year-old female patient came to our clinic with a personal history of rhinitis, umbilical hernioplasty, bilateral tubal ligation, bilateral mammoplasty, gluteoplasty, and ovarian cyst being followed up by gynecology and preeclampsia during pregnancy. She was referred to our service for cervical adenopathies of several weeks’ evolution. She denied any known trigger.

The exploration revealed several cervical adenopathies that had a major axis no more than two centimeters, were mobile and rubbery, and had no signs of extranodal extension. No skin lesions were found.

The initial study included general blood analysis with normal results and a rheumatologic screening, which was negative for all serologic markers such as rheumatoid factor, the human leukocyte antigen B27 (HLA-B27), and anti-cyclic citrullinated peptides (anti-CCP). Additionally, complement and inflammatory markers such as C-reactive protein (CRP) were all in the normal range.

**Figure 1:**
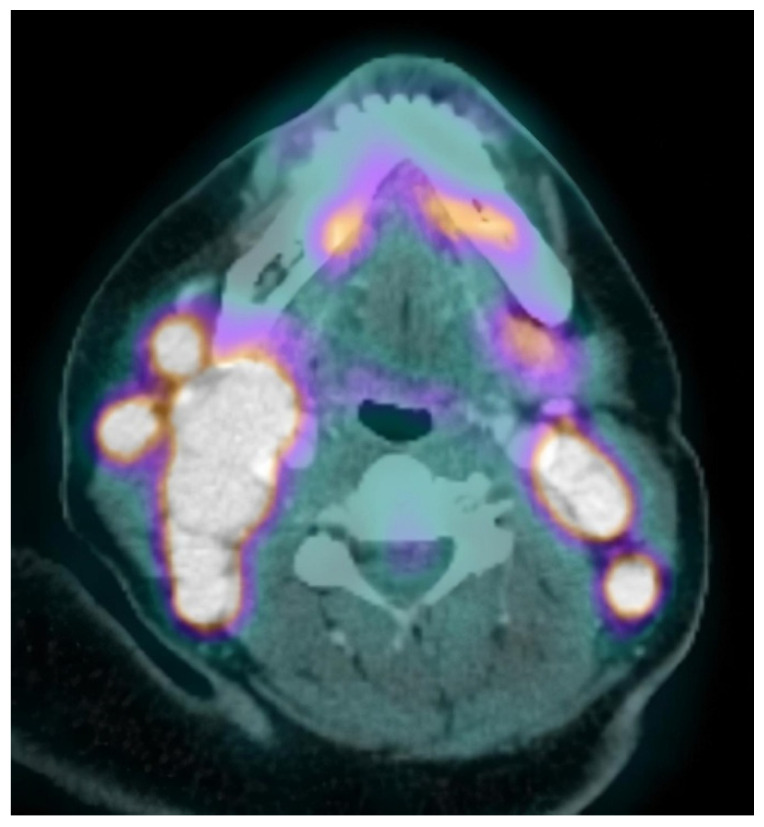
PET-CT: Pathological enhancement of several bilateral cervical lymphadenopathies.

Neck, chest, abdomen, and pelvis CT scans were carried out, showing several cervical, abdominal and pelvic adenopathies. In view of the diagnostic possibility of a lymphoproliferative syndrome, a surgical biopsy was performed on one of the cervical adenopathies with inconclusive results: non-specific mixed lymphoid hyperplasia (follicular and interfollicular), as well as the presence of abundant histiocytes with phagocytic cytoplasmic detritus. No neoplastic infiltrations were observed. Microbiological studies of the sample were negative. The battery of imaging tests was extended with a PET-CT, showing hypermetabolic lesions suggestive of malignancy in bilateral cervical and abdominopelvic adenopathies, subcutaneous tissue, and bone (Figure 1).

**Figure 2:**
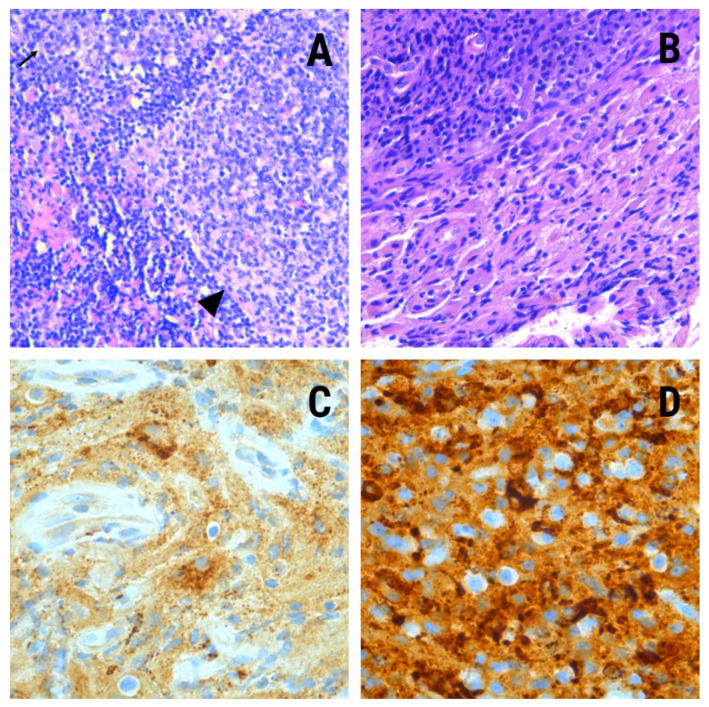
(**A**)—20×. Cervical lymph node. Arrowhead, germinal center; black arrow, histiocytes in interfollicular area. (**B**)—20×. Calcaneal bone. Histiocyte proliferation associated with mononuclear inflammatory elements such as plasma cells and lymphocytes. (**C**)—40×. Calcaneal bone. Blue staining corresponds to CD68. (**D**)—40× Calcaneal bone. Brown staining corresponds to CD163.

During hospitalization, the patient reported discomfort in the left ankle, which she related to an old sprain. Lower limbs MRI scan showed the presence of a left calcaneal injury with an increased signal from the fat marrow with well-defined edges and soft tissue edema. A biopsy was performed on this bone, whose pathological study showed histiocyte elements of non-Langerhans histiocyte IHQ profile: CD68(+)/CD163(+);/S100(−)/CD207(−)/CD1a(−) specifying the diagnosis of Erdheim–Chester disease (Figure 2).

Samples from the previous cervical adenopathy biopsy were reviewed, and immunohistochemical tests were performed on them, with identical results as on the calcaneal specimen. The mutational study for BRAFV600E was negative.

Once the disease was typed, a bone marrow biopsy was performed, in which hyperplasia of histiocytes with non-Langerhans IHQ profile was observed. Lumbar puncture and transthoracic echocardiogram were carried out with normal results. Brain MRI ruled out central nervous system (CNS) involvement.

Treatment with subcutaneous interferon-α (IFN-α) was started three times a week with acceptable tolerance. The patient presented a favorable evolution with partial metabolic response of cervical and abdominopelvic adenopathies and metabolic stability in skin and bone lesions.

## 3. Discussion

Erdheim–Chester disease (ECD) was described in 1930 by the Austrian pathologist Jakob Erdheim (1874–1937) and the American pathologist William Chester (1903–1974) as “lipoid granulomatosis” [1]. This disease is distinguished by xanthogranulomatous and xanthomatous infiltration of tissues with numerous spumous histiocytes—vacuolated appearance cell, loaded with lipids—surrounded by fibrosis in most cases [2]. To date, fewer than 1000 cases have been described in the scientific literature; it is therefore a strange entity [1,3]. The age of diagnosis is between 40 and 70 years. There is a predominance of male involvement. ECD has been classified for decades along with non-Langerhans cell histiocytosis. In 2012, advances in molecular techniques have made it possible to discover the presence of the BRAFV600E recurrent mutation in 40–80% of patients with ECD. Several other kinase mutations have also been described, and therapies targeting the BRAF and MEK pathways have been used with broad efficacy [5].

Mutations activating the MAPK pathway have an important role in the pathogenesis of ECD. The BRAFV600E mutation is linked to 7% of human cancers [6]. This oncogenic mutation allows the activation of the RAS-RAF-MEK cell signaling pathway, which is involved in functions such as apoptosis, angiogenesis, proliferation, migration, and cell survival. This is not the only signaling pathway involved in ECD, as other mutations activating the PIK3CA pathway have been found in up to 11% of diagnosed patients [1]. This is why therapies targeting the intracellular signaling pathways involved may be an effective treatment.

The organ damage caused by the disease is not the result of a proliferative mechanism; elevated C-reactive protein, a marker of systemic inflammation, is observed in more than 80% of patients. In addition, studies have demonstrated the inflammatory profile of ECD by detecting elevated levels of IL-8, which acts as a chemoattractant for polymorphonuclear cells and monocytes [7].

Bone involvement occurs in up to 96% of patients with ECD [1,2,3,4,5,6,7]. In fact, the diagnosis of ECD depends on a combination of clinical and radiological criteria, which are confirmed by histopathological findings [4]. In any case, only 50% of them describe bone pain. Another clinical spectrum includes multi-organ involvement, causing exophthalmos, papilledema, xanthelasmas, papulonodular skin lesions, diabetes insipidus, severe pulmonary disease, renal failure (perirenal fatty infiltration, known as hairy kidney), cardiomyopathy, and CNS disorders [1,2,3,4,5,6,7]. Due to its extensive and varied multi-organ involvement, today, PET-CT scintigraphy is vital for the evaluation of disease activity [2,3,4,5,6,7,8].

Nodal involvement has been described as an infrequent and uncharacteristic finding with other organ involvement. There are few published cases in which lymph node involvement associated with other organs has been identified but without being the initial cause of the clinical expression of the disease. This is the first reported case, to our knowledge, whose debut has been the appearance of cervical adenopathies. Histiocytes are found in numerous organs, including lymph nodes; although its involvement is common in Rosai–Dorfman disease, in this case, the cells were positive for CD68, CD163, and S100, which emphasizes the multisystemic character of Erdheim–Chester disease.

ECD is frequently associated with other systemic histiocytosis and auto-immune diseases. Twenty-one percent of patients present positive anti-double-strand DNA (dsDNA) antibodies and 12% anti SSA. Anti-phospholipid (APL) is also frequently seen with a predominance of anticardiolipidic antibodies. The most frequent auto-immune-associated diseases are auto-immune thyroiditis, primary Sjogren’s syndrome, and systemic lupus erythematosus [1,9].

The consensus for the diagnosis of ECD is based on histological findings together with clinical and radiological data appropriate to the context. Histological lesions are characterized by typical foamy infiltration of histiocytes surrounded by fibrosis in most cases [1,2,3,4,5]. Touton giant cells are also frequently present [8]. Immunohistochemical studies demonstrate histiocytes positive for CD68 and CD13. They are negative for CD1a, S100, and CD207. S100 positivity is observed sporadically. This differentiates ECD from Langerhans histiocytosis, in which the Langerhans cells are S100- and CD1a-positive (Table 1). Skeletal findings of bilateral and symmetric abnormalities are nearly always present in the diaphyseal and metaphyseal regions of the long bones of the legs, best visualized in femur and tibia [5,6,7,8].

The best imaging test for the study of the disease is, as mentioned above, PET-CT [1,2,3,4,5,6,7,8], although bone lesions can be seen on CT and MRI, assuming that they are often missed on plain films. There is a proportion of patients—around 4%—with an absence of bone lesions. In these cases, the diagnosis is based on histopathology and involvement of other organs. In either case, a biopsy is always necessary to confirm the diagnosis and to establish the presence or absence of the BRAFV600E mutation in order to focus treatment [1,8].

The differential diagnosis of ECD is compulsory because there are many conditions characterized by certain symptoms and findings similar to those commonly associated with ECD: Langerhans histiocytosis, Rosai–Dorfman disease (Table 1), Paget disease, amyloidosis, multiple sclerosis, neurosarcoidosis, metabolic diseases, mucopolysaccharidosies, Ormond’s disease, Gaucher’s disease, cerebrotendinous xanthomatosis, Wegener’s granulomatosis, Whipple’s disease, chronic recurrent multifocal osteomyelitis, vasculopathies, primary hypophysitis, cancers, and mycobacterial infections [2]. Histological examination of the biopsied lesions will serve to rule out all other clinical suppositions.

Referent to the treatment, there have been few prospective therapeutic studies and no randomized controlled trials in ECD [1,8]. It is generally assumed that initiation of treatment is better than clinical observation, with the rare exception of indolent patients [8]. The best therapy is interferon-α [1,10]. IFN-α has been identified as an independent predictor of survival in some retrospective studies, and it is the therapy with the largest amount of supporting evidence; its pegylated (PEG-IFN-α) formulation is recommended as first-line therapy and is preferred for the treatment of non-life-threatening manifestations of the disease. IFN-α/PEG-IFN-α has been found to have potential toxicities as fever, fatigue, flu-like symptoms, neurologic symptoms, gastrointestinal symptoms, alopecia, pruritus, transaminitis, and cytopenia [1,8].

The discovery that more than 60% of ECD patients have the BRAFV600E mutation led to the administration of Vemurafenib, a specific BRAF inhibitor, with robust responses in three patients [7]. Some clinical trials suggest that all patients with the BRAF mutation could benefit from treatment with Vemurafenib and should therefore be started as first-line treatment. However, response rates are variable, so treatment with Vemurafenib is often recommended as a second-line therapy or in the case of IFN failure or intolerance [1,8].

Imatinib mesylate has been successfully used for treatment of other histiocytic disorders. Results in seven patients treated with it have been variable, so it is considered a reasonable therapeutic strategy when first-line treatments have failed [8].

Anti-cytokine directed therapies such as Infliximab (targeting TNF-α) and anakinra (targeting IL-1β) are the most prescribed biotherapies. Anakinra seems to be more efficient with response rates from 22 to 57% but it appears to be less efficacious than IFN-α for patients with CNS or cardiac involvement [8]. In either case, disease progression rates are higher than the ones obtained with BRAF and MEK inhibitors. Tocilizumab (targets IL-6) seems promising but also is not recommended as first-line therapy [1].

Other treatments used in ECD include corticosteroid therapy, mainly for reduction of orbital edema in cases of exophthalmos but not useful for the treatment of the disease in monotherapy. Cladribine has also been postulated as a possible treatment, but low success rates have been reported. It may be considered as a second line of treatment [10].

The use of radiotherapy with bisphosphonates has been used to palliate bone pain. The role of surgical debulking is limited to urgent orbital decompression and resectable intracranial lesions [1,8,10].

Monitoring of the disease should be performed by FDG-PET every 3–6 months in patients starting treatment. Once the disease stabilizes, the time between scans may be extended. There are no disease-specific biomarkers; however, C-reactive protein levels may be useful for surveillance and monitoring the treatment.

Treatment is usually prescribed indefinitely, but long-term use of BRAF inhibitors may be detrimental due to the potential development of premalignant lesions [10].

The prognosis of ECD is relatively poor, with a survival of 43% of patients after 32-month follow-up. Survival rates of 68% at 5 years have been reported in patients treated with interferon [10].

## 4. Conclusions

ECD is a multisystem disease with an uncertain prognosis in which early diagnosis by immunohistochemical techniques and PET-TC is crucial for initiation of treatment. Knowledge of this entity is fundamental in the differential diagnosis in the context of diseases debuting with adenopathies in the head and neck area. Moreover, communication between the surgeon and the pathologist is essential for clinical guidance in infrequent and complex diagnostic cases.

## Figures and Tables

**Table 1:** Comparison between Erdheim–Chester disease (ECD), Langerhans cell histiocytosis (LCH), and Rosai–Dorfman disease (RDD) findings.

	ECD	LCH	RDD
Organ Involvement			
Skin	Yellow plaques Xanthelasma,	Scaly erythematous patches	Indurated papules
Bone	Femur and tibia. Bone pain	Craniofacial bones, proximal limbs, pelvis, scapula	Uncharacteristic
Heart	Pericardial effusion, myocardial infiltration, etc.	Uncharacteristic	Uncharacteristic
Liven and spleen	Rare	Uncommon but constitutes high-risk disease	Uncharacteristic
Lungs	Interlobular septal thickening, ground-glass or centrilobular opacities on CT	Nodular and cystic changes in upper and middle lobes	Uncharacteristic
Lymph nodes	Uncharacteristic	Uncommon but constitutes high-risk disease	Cervical LN, axillary, inguinal, paraaortic or mediastinal
Retroperitoneum	Perinephric infiltration	Uncharacteristic	Uncharacteristic
CNS	Dural lesions Brain parenchimal lesions	Dural lesions Brain parenchimal lesions	Dural lesions
Histopathologic Features			
CD68	+	+	+
CD163	+	+	+
CD1a	−	+	−
CD207	−	+	−
S100	−	+	+
Touton giant cells	+	−	−
Other Characteristics	Xanthomatous features, fibrosis	Birbeck granules on electron microscopy	Intracytoplasmic lymphocytes (emperipolesis)

## Data Availability

The data presented in this study are available on request from the corresponding author.
